# Practical Medicine

**Published:** 1876-05

**Authors:** 


### II. Practical Medicine.

1. *Fatty Degeneration of Liver and Kidneys Consecutive to an Extensive Burn.* Couston. (*Le Progrès Méd.*, No. 9.)

A patient (male, 27 years old, who had enjoyed previous health without syphilitic, scrofulous, tuberculous or alcoholic antecedents), in consequence of an accident with a kerosene lamp, was extensively burned upon the posterior part of the trunk, from the lumbar region to base of the mucha. The pain, excesssive at first, was partially relieved on the second day afterward. There was no cerebral excitation, only moderate fever with anorexia, dyspnœa and dysuria, with a red and very scanty urine. A persistent diarrhœa supervened, without colic or abdominal pain. The abdomen remained supple, and there was no blood in the stools. Then followed profuse sweating, separation of sloughs, extensive suppuration of the burned surface, formation of eschars over the trochanters and shoulder blades, whose fall left bare the articular capsules, great prostration, and finally death.

At the autopsy the lungs were found intensely congested, and the liver enormously hypertrophied and fatty in all parts save in the lobus Spigelii. The renal substance was also in a granulo-fatty degeneration.

The femoral and external iliac veins of the left side were filled with a clot, which terminated exactly where the primitive iliac vein arises. In detaching the femoral vein, some collateral branches were divided, which gave exit to a drop of pus. These vessels were buried in the muscles of the external region of the thigh. It was difficult to decide whether they communicated with the trochanteric and sacral abscesses. In the femoral vein, at the points where the latter opened, were small purulent foci, perfectly encysted and separated from each other by portions of clots, which were exclusively fibrinous.

Duret remarked that venous thrombosis following a burn was rare, but had yet been noted (Guy's Hospital Reports). He had recently seen peculiar cerebral phenomena after a severe burn of the surface of the chest. There was undisturbed intelligence, but aphasia and hemianæsthesia, which disappeared subsequently, and which he was disposed to refer to encephalic thrombosis.

2. *Mumps: its Relation to the Eruptive Fevers.* COLIN. (*La France Méd.*, No. 18.)

From the comparison instituted it is evident that: (*a*) mumps and the exanthematous fevers have the same mode of transmissibility; (*b*) immunity conferred by a first attack; (*c*) no sporadicity; (*d*) frequency among children and soldiers; (*e*) slow and successive spread among various classes of people, when the element of time cannot contribute an appreciable influence—the disease seeming to lose its power in one situation before its extension to another; (*f*) but the point of contact between mumps and the eruptive fevers seems to be the simultaneity of the epidemics of the two—among the last named especially measles, which seems to precede or accompany mumps.

There seems to be likewise a species of affinity between these disorders, so far as regards the conditions which produce the "medical constitutions" in each.

A clinical resemblance may be discovered in the period of incubation and invasion, in the febrile movement, and the secondary fever which ushers in metastatic orchitis. It is true, however, that in adults the affection may be initiated by a period of calm, which does not recall the febrile movement in the pyrexias, and also that the involvement of the testicle, primary or secondary, may be so sudden and unexpected as to suggest traumatism or a venereal affection. There are other cases in which the general symptoms are severe: typhoid symptoms, convulsions with a fatal termination, intense articular pains, an exanthema or enanthema of variable degree, anasarca, with albuminuria. Colin cites cases where the latter complication has been noted, and where the metastasis has been to the kidneys instead of the testes. One fatal case suffered from orchitis, acute albuminuria and uræmia.

3. *Antagonism between Heart Disease and Tuberculosis.* PETER. (*Gazette des Hôpit.*, Nos. 97 and 99.)

There is no such absolute antagonism. Tubercles develop by preference in the rudimentary tissues (connective), and in organs or parts of organs of minimum function. Thus it is that tubercles are found at the pulmonary apices, the parts of the lung which have the least work to do because of the shortness of the upper ribs, their relative immobility, and the course of the superior bronchi, relieved by the current of air from the sense of weight. In mitral affections the inferior portions of the two lungs are, at a certain period, in a stage of chronic congestion. It results from this that the upper part of the lungs, in order to perform a supplementary labor in hæmatosis, rendered necessary by this congestion, are compelled to do extra work. From this functional hyperactivity it happens that the pulmonary apices have a diminished tendency to become tuberculous. This explains the pretended antagonism between the two disorders.

But, at the onset of mitral insufficiency, when there is less interference the circulation, and the inferior lobes are not yet affected with passive congestion, the superior lobes are then not in such a condition of exalted functional activity as to make immunity probable. Tubercles may then be developed if the patient is placed under such conditions as will ordinarily favor this process—bad hygiene, poverty, etc.

There is, therefore, no absolute immunity of the pulmonary apices for tuberculosis when there is coexistent disease of the heart. All is subordinate to the period of the cardiac disease, to the nature of the latter, and to the general condition of the organism.

(We were recently consulted by a gentleman who was suffering from mitral insufficiency with a loud systolic murmur, who had had several hæmoptyses, and unmistakable evidence of tuberculosis of the right pulmonary apex.—Ed.)

4. *Thoracic Hyperæsthesia and its Relation to Acute Tuberculosis.* Bouchut. (*Gazette des Hôpit.*, No. 64.)

B. discovered in a child, who presented the evidences of a typhoid fever, tubercles of the choroid, the signs of an acute tuberculosis. There was also thoracic hyperæsthesia. This last symptom, which he has several times observed, should be added, he thinks, to the list of symptoms of acute tuberculosis. It points unmistakably to a lesion of the pleura which autopsies have demonstrated. This hyperæsthesia is not to be found in typhoid fever, for, when discovered, it is in every case not limited to the chest, but has extended to it, to the membranes, etc. The same is true of the tegumentary hyperæsthesia of tuberculous meningitis. Therefore whenever, in a disease of a typhoid type, there exists a thoracic hyperæsthesia, not complicated by hyperæsthesia of the limbs, it indicates dry pleurisy and acute pulmonary tuberculosis.

5. *Progressive Muscular Atrophy.* Wm. Alexander, M.D. (*Lancet*, March, 1876.)

Dr. A. records five cases of this disorder, with comments as to its nature. The cases resembled each other in having nervous symptoms at the beginning, in the onset being gradual, in the upper extremities being principally affected, in the absence of any hereditary quality or any specific taint, in the slight improvement following the constant current and shampooing, and in the irregular manner of the advance of the disease. The author believes the disease has a nervous and not a muscular origin, and for the following reasons: 1, The nervous symptoms at beginning—a feeling of "pins and needles," neuralgic pains, numbness and susceptibility to change of temperature in the affected part. 2, On the muscular theory the course of the disease is inexplicable—it invades one hand, passes up the arm for some distance, then attacks the opposite hand; it attacks one bunch of muscles, and passes by another bunch in close contiguity. "On the theory that it is a disease of the cord, its course is just what we ought to expect." 3, The treatment that was most beneficial was such as would, we might suppose, have made the disorder worse had its origin been muscular and not nervous. 4, The *post mortem* made with microscopic examination of the cord showed the disease in this structure to be far in advance of the change in the muscles—the reverse ought to be true did the affection begin in the muscles. 5, The fact that spinal cords taken from patients who had died of the disease have, on a rough examination, been thought to be healthy, does not negative the theory of nervous origin.

6. *On Unique Anatomical Appearance in General Paralysis.* W. H. DeWitt, M.D. (*Cincin. Lancet and Obs.*, April, 1876.)

The history of this case offered nothing unusual for a case of general paralysis of the insane. Death occurred in about a year from the beginning of the disorder. During the early months there was the usual exaltation and vast "intellectual pretensions," with great irritability of temper. The temperature "was above the normal during the whole course of the disease."

The dura mater was found much thinner than normal, and presented at points a cribriform appearance. Between the visceral and parietal layers of the arachnoid, there was a false membrane that enveloped the entire brain structure. "It was but slightly adherent, and easily detached without destroying its texture." The arachnoid was opaque, and the pia mater was only slightly congested. The convolutions were flattened and the cortical substance was thin.

Dr. D. believed the thinning of the dura mater was due to the pressure by the false membrane, and that the latter was an appearance not previously reported.

7. *Intussusception in an Infant—Recovery.* F. L. Haynes, M.D. (*Phila. Med. Times*, March 18, 1876.)

The patient was seven months old, and had always been constipated. On symptoms of intestinal disturbance, castor oil was given, which operated well. Diarrhœa followed—in two days streaks of blood characterized it. The next day there were more blood, mucus and tenesmus ; injections of starch and laudanum, and stoppage of diarrhœa, with beginning of vomiting of greenish fluid. The day following came stercoraceous vomiting, tympanitis, dullness on percussion, and tumefaction over descending colon, with great prostration. A tumor could be felt by the finger in the rectum, a soft, velvety mass filling the whole space, and having an oblong orifice in the centre. The child was suspended by his heels, the tube of a stomach pump was pressed far into the rectum, and water forced in by means of a Davidson syringe. After repeated attempts no good was accomplished. Later in the day another attempt was made in the same way, when the tumor disappeared. The child now made a slow recovery. Flatus was at once evacuated by passing a tube into the rectum.

8. *Intra-Cranial Aneurism—Recovery Under Large Doses of Iodide of Potassium.* Wm. E. Humble, M.D. (*Lancet*, March, 1876.)

This is a case previously reported of an aneurism in the cavernous sinus. For seven and one-half months the bruit was so distinct that the patient constantly heard it, and was much annoyed by it. During five months Dr. H. was able to hear it with the stethoscope. Three and one-half months after the first symptoms appeared, the case was put upon the regular use of the iodide. Most of the time thirty-six grains were taken daily. The general health during the four months of the administration of the drug continually improved. At the end of this time the bruit suddenly stopped. The patient had some neuralgia and other unpleasant symptoms of lesser moment, but at the end of five weeks there had been no return of the bruit, and the aneurism appeared to be cured.

9. *Paracentesis of the Pericardium—Recovery.* Dr. Burder. (*Lancet*, March, 1876.)

A carpenter, sixty years old, had had, forty years before, and again two years later, two attacks of rheumatic fever. Subsequently, and until about Christmas, 1874, his health was good. At the last-named date he was attacked again with rheumatism, which kept him in bed a week. In April, 1875, after having had a cough for some days, his feet began to swell.

He was found, April 26th, with general anasarca, shortness of breath and feeble pulse. The heart's action was rapid, irregular, with an indistinct systolic bruit. Two days later he was apparently moribund. The signs indicating considerable fluid in the pericardium, he was aspirated, and forty-two ounces of the material—pale, straw-colored—removed. The needle was inserted between the 5th and sixth ribs, and one inch to the right of the nipple. Toward the close of the operation the apex of the heart was felt to strike the needle once or twice, but it ceased on placing the needle more horizontally. The patient was easier immediately after the operation; in a few hours he was very much easier. Recovery resulted.

10. *The Relation of the Teeth to Disturbances of the Nervous System.* (*The Dental Register*, March, 1876.)

The following cases are reported:

A woman, unmarried, twenty-five years old, healthy, and with teeth better than the average, had at times, for months, paralysis of the left arm. After receiving a variety of treatment without avail, her teeth were examined by a dentist, when the nerve of one of the wisdom teeth was found to be bare. The tooth was extracted, when the palsy was cured.

A woman of good health, aged twenty years, was married; soon after which she had epilepsy. She had treatment, but grew worse for three years, when, on advice, she had all her diseased teeth extracted; this included all except the eight front ones of the lower jaw. She took tonics and was well.

A married woman of twenty-five, had never borne children. Her teeth and other appendages were perfect. She had toothache regularly at her menstrual periods.

